# A prospective analysis of the correlation between ultrasonic B-lines, cardiac tissue doppler signals and left ventricular end-diastolic pressure in patients with severe aortic stenosis

**DOI:** 10.1186/s44156-024-00055-y

**Published:** 2024-08-12

**Authors:** Zouheir Ibrahim Bitar, Ossama Maadarani, Hussien Dashti, Abdullah Alenezi, Khaled Almerri

**Affiliations:** 1 Consultant critical care medicine Internal medicine department Ahmadi hospital, Ahmadi, Kuwait; 2 Critical care Unit, Ahmadi hospital Kuwait oil company, Ahmadi, Kuwait; 3 Consultant internal medicine Internal medicine department, Ahmadi hospital, Ahmadi, Kuwait; 4 Consultant interventional cardiology Cardiology depart, Chest Hospital, Shuwaikh, Kuwait; 5 Department of Cardiology, Chest Disease Hospital, Shuwaikh, Kuwait; 6 Critical care Unit, Ahmadi hospital, Fahahil, 64015 Kuwait

**Keywords:** Lung ultrasound, Left end-diastolic pressure, B-lines, Aortic stenosis

## Abstract

**Background:**

The development of heart failure is a turning point in the natural course of aortic stenosis (AS). Pulmonary oedema and elevated left ventricular pressure (LVP) are cardinal features of heart failure. Evaluating pulmonary oedema by lung ultrasound involves taking the upper hand with a bedside noninvasive tool that may reflect LVP.

**Aim:**

We sought to assess the correlation between sonographic pulmonary congestion, invasive LV pre-A pressure, and echocardiographic LV end-diastolic pressure (LVEDP) in symptomatic AS patients receiving transcatheter aortic valve replacement.

**Methods:**

Forty-eight consecutive patients with severe AS and planned transcatheter aortic valve implantation (TAVI) were enrolled. LVEDP was estimated to be normal or elevated using the ASE/EACVI algorithm and transmitral Doppler indices, the E/A ratio, the E/e′, and the left atrial volume index. Invasive LV pre-A pressure was used as a reference, with > 12 mm Hg defined as elevated.

**Results:**

Forty-eight patients (25 women (52%), mean age 75 years, standard deviation (SD) ± 7.7 years) were enrolled in the study. We detected severe B-lines (≥ 30) in 13 (27%) patients and moderate B-lines (15–30) in 33 (68.6%) patients. The number of B-lines increased significantly with the severity of New York Heart Association (NYHA) functional classes (Fig. 1). The B-line count was 14 ± 13 in NYHA class I patients, 20 ± 20 in class II patients, and 44 ± 35 in class III patients (*p* < 0.05, rho = 0.384). The number of B-lines was correlated with the E/E’ ratio (*R* = 0.664, *p* < 0.0001) and the proBNP level (*R* = 0. 882, *p* < 0.008). We found no significant correlation with the LVEDP or LVEF. The LVEDP correlated well with the E/E’ ratio (*R* = 0.491, *p* < 0.001) but not at all with E/A, DT, or LAVI. All patients had an elevated LVEDP > 12, with a mean pressure of 26 mmHg, a minimum of 13 mmHg, and a maximum of 45 mmHg, with an SD of 7.85.

**Conclusion:**

Assessing lung ultrasonic B-lines is a straightforward and practical approach to identifying pulmonary oedema in AS patients. The number of B-lines correlated with the E/E’ ratio and the functional status of patients but did not correlate with invasive LVEDP or LVEF. All patients had elevated LVEDP that correlated with E/E’.

## Introduction

*Aortic stenosis* (AS) is a common degenerative valve lesion requiring surgical transcatheter intervention in the Western world [1]. Its prevalence is rising rapidly due to the ageing population [2]. Heart muscle scarring and fibrosis are key factors that worsen left ventricular function in patients with aortic valve disease, regardless of the presence or absence of coronary artery disease; these conditions can be diagnosed and quantified using cardiac magnetic resonance. Amyloidosis is also recognized to be associated with AS in elderly patients (incidence 9.15%) [3]. However, the correlation between AS severity and the onset of symptoms and their severity is poor. The left ventricle compensatory response to pressure afterload is the primary determinant [4]. As a result, the values calculated from echocardiography findings classifying aortic stenosis severity are not predictive of the onset of symptoms.

On the other hand, patients with even moderate AS may also experience symptoms, and recent literature suggests that the prognosis is not benign and may benefit from early aortic valve intervention [5]. The definition of severe aortic stenosis is highly sensitive but not specific; many patients who meet the definition of severe AS are not symptomatic [6]. However, when severe AS is present, symptomatic patients require intervention because average survival without intervention is only two to three years, and these patients have a high risk of sudden death [6].

Pulmonary congestion and oedema are frequent and can result in heart failure. It is presented as an ultrasonic B line in lung ultrasound (LUS). Evaluating B-lines is a noninvasive, radiation-free, and semiquantitative tool for assessing pulmonary oedema at the bedside [7, 8]. B-lines can anticipate elevated Pro-BNP and reflect elevated LV and pulmonary capillary wedge pressure [9]. This feature of a noninvasive tool correlates well with the degree of extravascular lung water and pulmonary capillary wedge pressure in heart failure patients [10, 11]. Furthermore, LUS can detect clinically silent pulmonary oedema [10], suggesting that it can be used to assess haemodynamics and optimize treatment [8]. In the present study, we investigated the correlation between different diastolic function parameters and invasive LVEDP in patients with severe AS prepared for TAVI. The subanalysis included the correlations between B-lines, LVEDP, and pro-BNP in the same population.

### Setting and study population

This observational, prospective study was conducted at the cardiac centre of the chest hospital and Ahmadi Hospital, Kuwait, from May 21 to October 2022.

## Methodology

### Echocardiography

Consecutive patients with severe AS referred for chest disease and TAVI were registered. The main inclusion criterion was as follows: 1) severe echocardiographic AS identified by an aortic jet velocity greater than ≥ 4.0 m/s or a mean transvalvular pressure gradient ≥ 40 mmHg as well as the typical appearance of a valve with severely reduced leaflet opening; an aortic valve area (AVA) ≤ 1.0 cm2 is typically observed but was not required to identify high-gradient severe AS.

The study excluded patients with low-flow low-gradient AS (mean gradient < 40 mmHg, AVA > 1 cm2, LVEF < 50%), associated moderate or severe aortic regurgitation, sequential moderate or severe mitral regurgitation, or severe, decompensated HF requiring urgent hospitalization (NYHA class IV). Echocardiography is an indirect method for evaluating left ventricular filling pressure (LVFP), which reflects myocardial relaxation and stiffness in patients with diseases of the LV [12, 13]. The early mitral annular velocity (e’) measured by tissue Doppler imaging indicated relaxation of the left ventricular myocardium. The ratio of the transmitral peak early filling velocity (E) to e’ (E/e’) is used as an indirect measure of left ventricular end-diastolic pressure (LVEDP). An E/e’ lateral > 12, an E/e’ mean > 13, or an E/e’ septal > 15 indicates elevated LVFP, while an E/e’ < 8 at any location indicates normal LVFP [6].

To measure the E and E’ velocities during both systole and diastole, a 5-mm sample volume was placed over the lateral or medial part of the mitral annulus from the apical four-chamber view. This showed the movement of the mitral annulus along its length. The velocity scale was adjusted to approximately 20 cm/sec on both sides of the zero-velocity baseline. To minimize the angulation between the plane of cardiac motion and the ultrasound beam, we reduced it to the minimum. The recommended sweep speed for spectral recordings is 50 to 100 mm/sec at end-expiration [13].

All reported echocardiographic measurements were taken as the average of three consecutive cycles. Additionally, the left ventricular (LV) volume and LV ejection fraction were assessed according to the recommendations of the American Society of Echocardiography (ASE) [10]. In addition, the mitral inflow was analysed for peak E (early diastolic) and peak A (late diastolic) velocities, E/A ratio, and deceleration time of the E velocity.

Patients with ischaemic heart disease were defined as patients with chronic coronary syndrome according to ESC guidelines [14]. Renal impairment and chronic kidney disease were defined based on the presence of either kidney damage or decreased kidney function for three or more months irrespective of cause [15].

All patients were evaluated in the echocardiography department of the chest hospital as a preoperative assessment before surgery. A Vivid-S70 (GE Vingmed, Horten, Norway) echocardiology machine was used. Patients were in stable ambulatory condition. Before inclusion in the study, the patients signed an informed consent form. The handling and publication of the data were in accordance with the Declaration of Helsinki. The ethical approval registration number is 2019/1108 from the Ministry of Health, Kuwait.

### Lung ultrasound

**Fig. 1:**
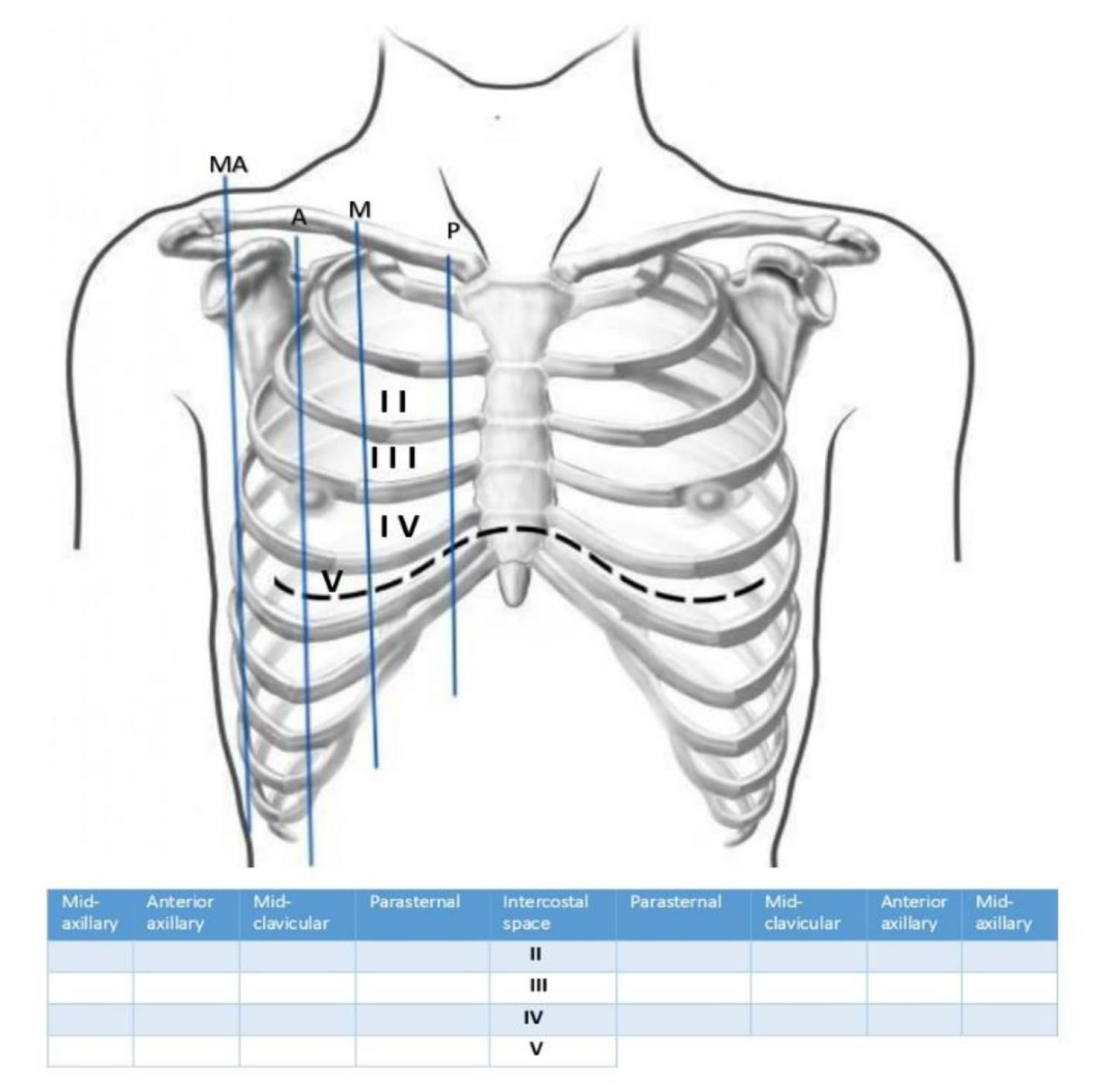
Flow chart. Method of B-line quantification. Twenty-eight scanning site scheme of the antero-lateral chest; P, parasternal; M, midclavicular; A, anterior axillary; MA, midaxillary

Lung ultrasonography was performed for every patient admitted to the cardiology ward for TAVI. The front and side parts of the hemithorax were scanned by moving the probe along four different lines on each side (parasternal, midclavicular, anterior axillary, and mid-axillary lines), from the second to the fifth intercostal space on the right and from the second to the fourth rib on the left. Twenty-eight spots were checked in total following a method that was previously described [16] (Fig. 1 ).A senior intensivist who was certified in critical care ultrasound examined the ultrasound images that were stored on a hard drive. Ultrasound imaging was performed with a portable ultrasound machine (GE Vivid iq, Horten, Norway) that had a curvilinear transducer with a 3.5-MHz broadband.

### Measurements

**Fig. 2:**
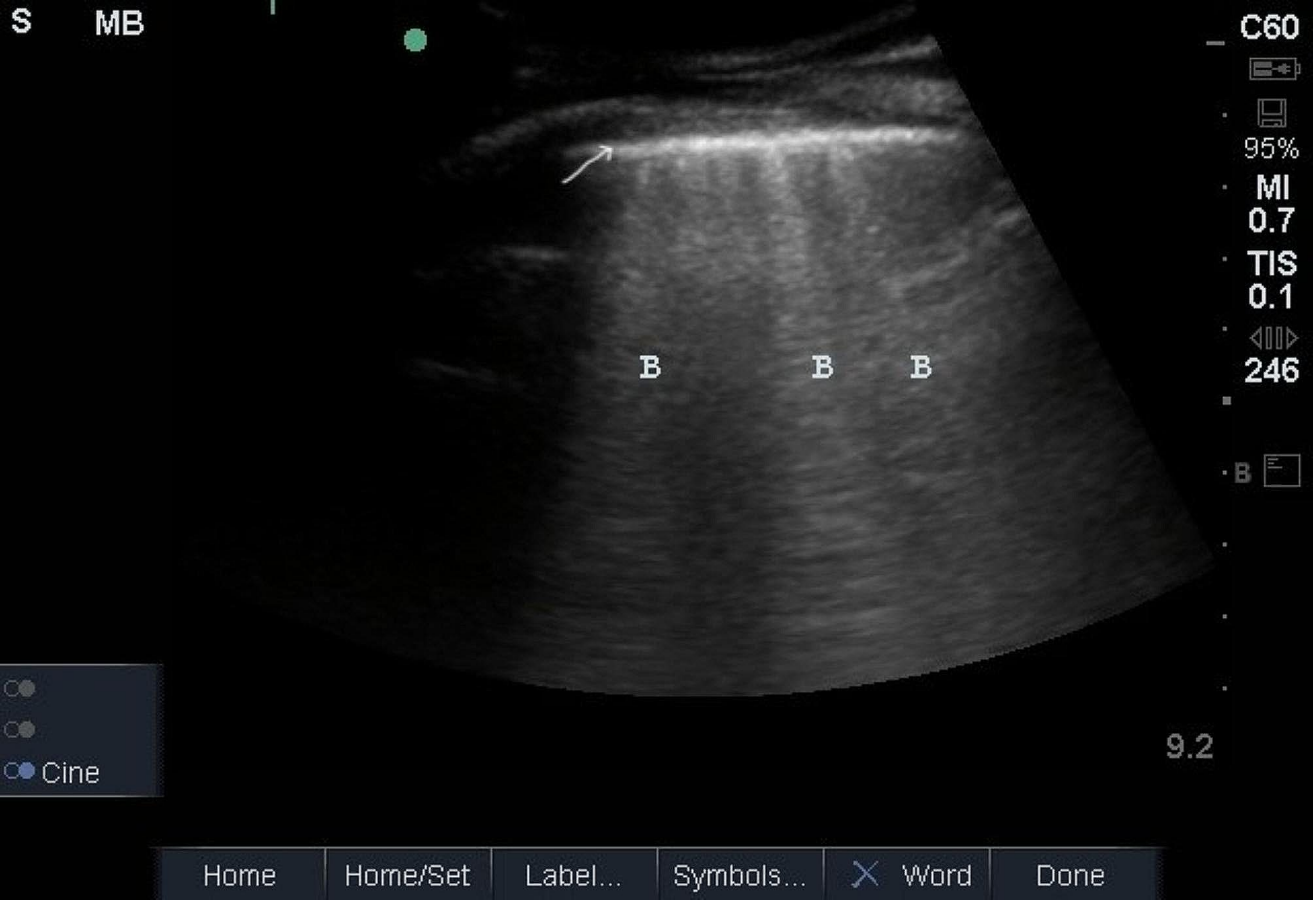
Example of a frozen loop of lung ultrasonography with three B-lines (B);, arrow showing the pleural line

**Fig. 3:**
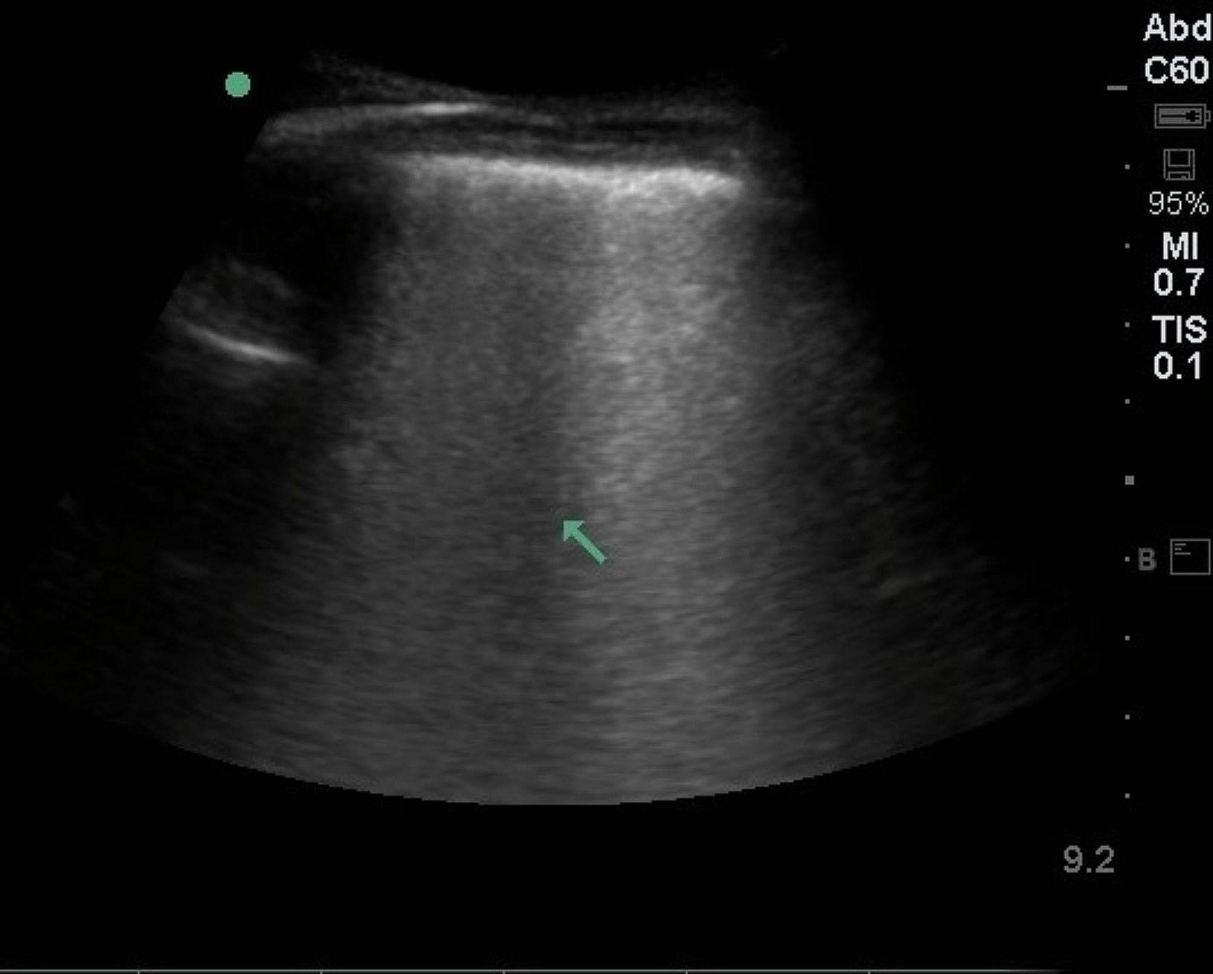
Example of a frozen loop of lung ultrasonography with extensive nine B-lines (arrow);

On ultrasound, normal healthy lungs show pleural sliding and A-lines (repetitive lines parallel to the pleural line) [6]. Multiple B-lines (more than three lines in one region) indicate interstitial syndrome (Figs. 2 and 3). After admission for TAVI, patients underwent a B-line assessment. The number of B-lines was counted for each spot. If they were separated, we recorded the total number (up to 10 for each site); if they were merged, we estimated the percentage of the screen they filled and divided that by 10 [16]. A total B-line score of 30 or more indicated severe pulmonary congestion [16].

### Cardiac catheterization

Before the TAVI procedure, angiography was performed, and the wire was inserted across the AV to measure the LVEDP. The LVEDP is conventionally measured at the Z-point (coincident with the R-wave on the corresponding ECG), and the LVEDP immediately precedes the beginning of isovolumetric ventricular contraction on the left ventricular pressure waveform (“Z” point). This point is situated on the downslope of the left ventricular “a” wave and marks the crossing over of left atrial and left ventricular pressures; this point is identified on the left ventricular pressure tracing as the point at which the slope of the ventricular pressure upstroke changes, which coincides with the R wave on the electrocardiographic tracing.

### Statistical analysis

Continuous variables are presented as either the mean ± standard deviation or median and interquartile range, as appropriate for each patient. One approach was to use the standard normal distribution formula, which is Z-Score = (X - µ) / σ, where X is a normal random variable, µ is the average or the mean, and σ is the standard deviation. The t test and Chi-square test were used for categorical data. A t test was used to compare the means between two groups to determine if they were significantly different from each other.

The Chi-square test was used to determine if there was a significant association between two categorical variables. A *p* value of less than 0.05 was considered indicative of statistical significance, meaning that the results were considered statistically significant if the probability of obtaining them by chance was less than 5%. To assess the correlations between parameters, parametric Pearson or nonparametric Spearman correlation coefficient analysis was used as appropriate. The data analysis was performed using IBM SPSS 19 statistical software.

## Results

Sixty-two patients were selected from May 21 to October 2022. Forty-eight patients (25 women (52%), mean age 75 years, SD ± 7.7 years) were enrolled in the study.

Table 1 shows the initial characteristics of the study population and comparisons between the different severities of ultrasound B-lines.

**Table 1:** Clinical characteristics of the patients in relation to ultrasound chest profiles

	All	severe degree of B-lines (≥ 30) in [13]	moderate (15–29) in 33 68.6%	Mild < 15	*P*
Age	75 ± 7.7	78 ± 4	76 ± 6	72	0.24
Sex (female) (%)	25 (52%)				
BMI	27.11 ± 3.8	28.22 ± 3.4	26.99 ± 4.19	25.84 ± 5	0.812
SBP (mmHg)	132.82 ± 12	140.85 ± 11	134.00 ± 14.7	127 ± 80	0.521
DBP (mmHg)	76.66 ± 8	76.71 ± 6	76.71 ± 10.8	66.8	0.977
NYHA1	13 ± 12	0	0	13	
NYHA2	19 ± 15				
NYHA3	43 ± 34				
DM	37	9	27	1	0.45
hypertension	45	12	31	2	0.54
Furosemide (%)	2 (4.2)	1	1	0	0.4
Haemoglobin	13 ± 2	11 ± 3	14 ± 1	12 ± 2	0.88
Renal impairment (%)	24	8	15	1	0.4
Haemodialysis no (%)	4	2	2	0	0.5
IHD (%)	34 (70)	10	22	2	0.56
LVEF (mean) (%)	60.23	44.6	43.48	56	0.346
CABG (no)	34	10	22	2	0.56
Invasive LVEDP	28.3	30.00	26.46	25.5	0.001
E/E’	20.59	25.53	18.87	12	0.001
DT	228.5	209	221	240	0.168
E/A	1.6	1.5	1.11	0.85	0.293

BMI, body mass index; SBP, systolic blood pressure; DBP, diastolic blood pressure; NYHA, New York Heart Association functional class; DM, diabetes mellitus; IHD, ischaemic heart disease; LVEF, left ventricle ejection fraction; CABG, coronary bypass graft; LVEDP, left ventricle end-diastolic pressure; E/E’, ratio between early mitral inflow velocity and mitral annular early diastolic velocity; DT, deceleration time; E/A, ratio between the E-wave and A-wave

**Fig. 4:**
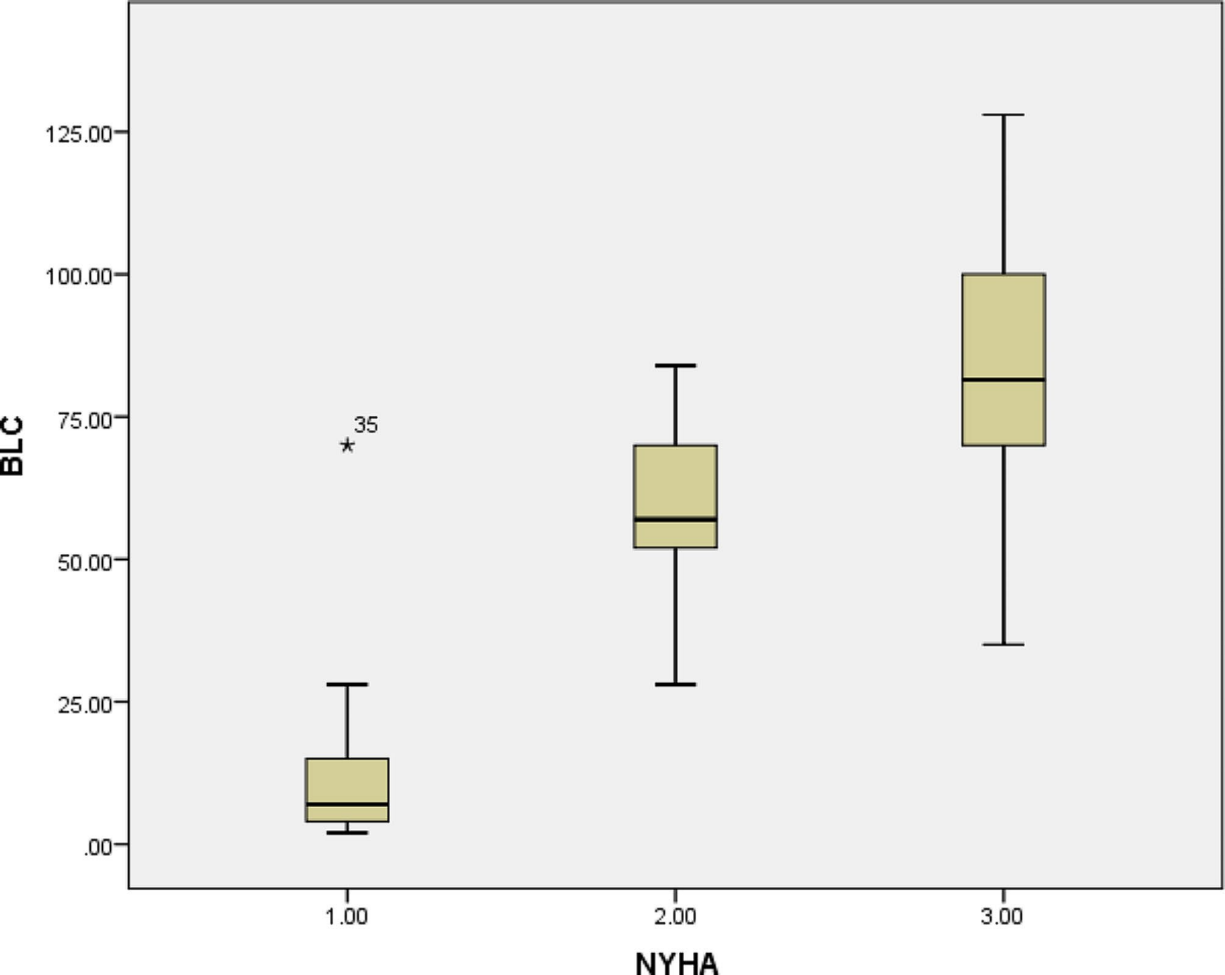
Increasing number of B-lines with worsening NYHA functional class NYHA, New York Heart Association; BLC, B-line count

We detected severe B-lines (≥ 30) in 13 (27%) patients and moderate B-lines (15–30) in (33) 68.6% of all patients. The study showed that the number of B-lines increased significantly with the severity of New York Heart Association (NYHA) functional classes (Fig. 4). The B-line count was 14 ± 13 in NYHA class I patients, 20 ± 20 in class II patients, and 44 ± 35 in class III patients (*p* < 0.05, rho = 0.384).

**Fig. 5:**
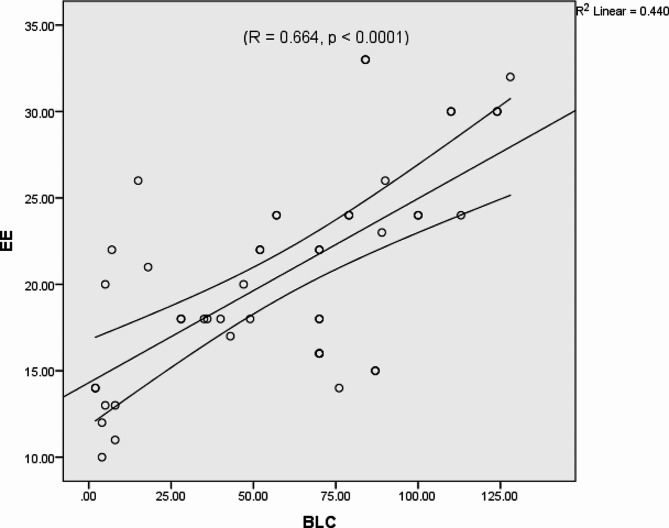
Correlation between B-line counts (BLCs) and tissue Doppler (EE) Doppler signals The standard R function for calculating Pearson correlation

**Fig. 6:**
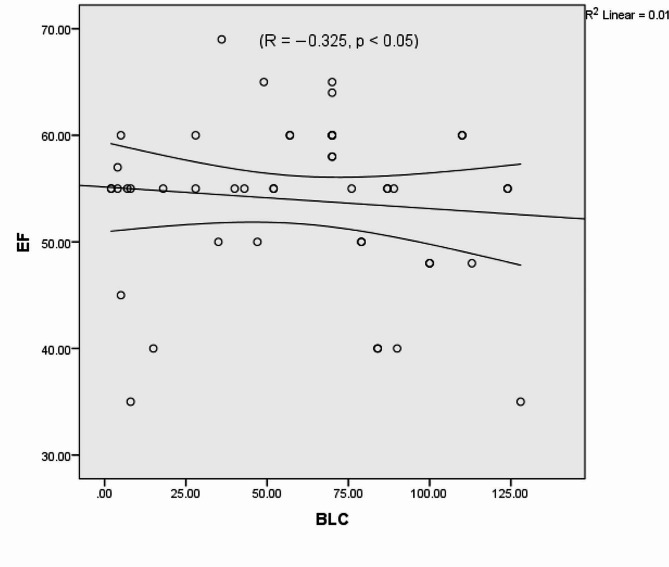
Poor correlation between the measured ejection fraction (EF) of the left ventricle and B-line count (BLC) The standard R function for calculating Pearson correlation

**Fig. 7:**
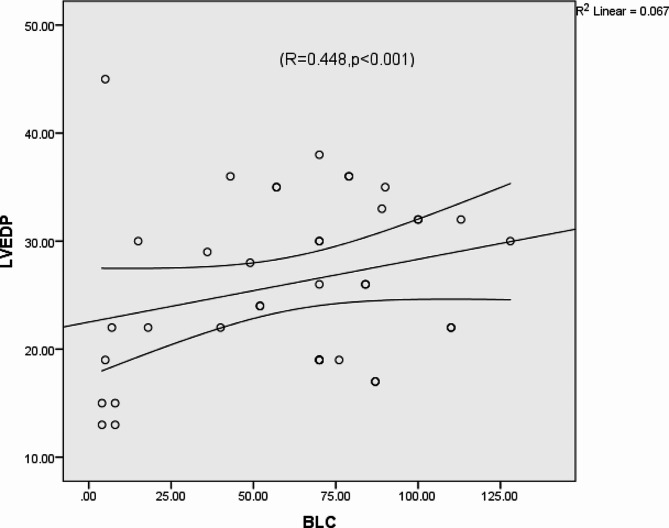
Direct left ventricular end-diastolic pressure (LVEDP) in correlation with tissue B-line count (BLC) The standard R function for calculating Pearson correlation

The number of B-line artefacts was correlated with the E/E’ ratio (*R* = 0.664, *p* < 0.0001) (Fig. 5), and the pro-BNP level (*R* = 0. 882, *p* < 0.008) but did not correlate with left ventricular EF or LVEDP (Figs. 6 and 7). We found no significant associations between E/A or DT and LAVI.

**Fig. 8:**
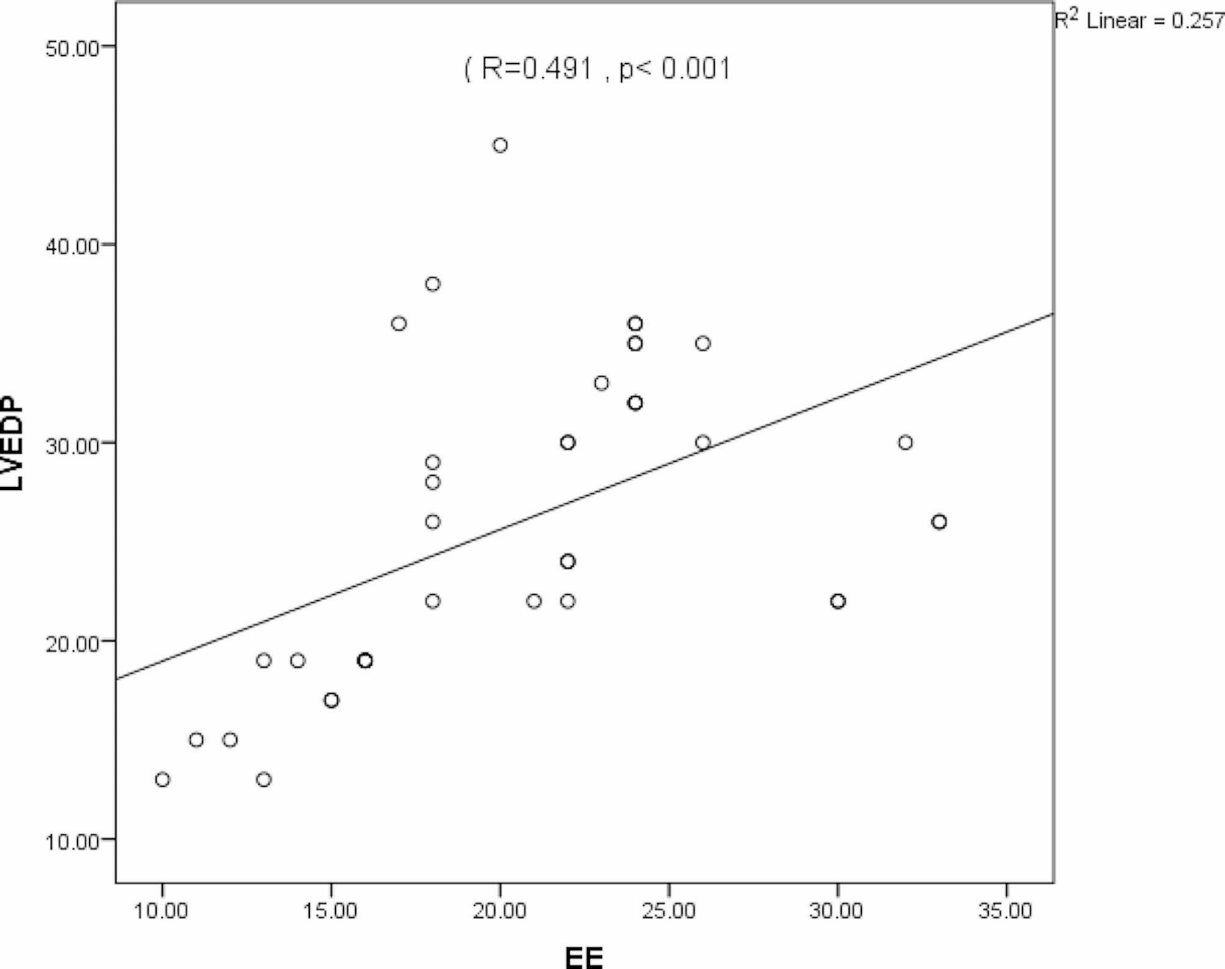
Direct left ventricular end-diastolic pressure (LVEDP) as a function of tissue Doppler (EE) The standard R function for calculating Pearson correlation

The LVEDP correlated well with the E/E’ ratio (*R* = 0.491, *p* < 0.001) (Fig. 8). However, there was no correlation with E/A, DT, or LAVI.

All patients had elevated LVEDP > 12, with a mean pressure of 26 mmHg (SD 7.9).

Fourteen patients were excluded from the initial population; 8 patients had concomitant multiple valvular heart disease (four patients had significant aortic regurgitation, and four patients had significant mitral regurgitation). Four patients had moderate AS associated with dilated cardiomyopathy, and another four patients had low-flow low-gradient AS. One patient was excluded because of active lung cancer, and three patients had severe obstructive airway disease as documented by pulmonary function tests done preoperatively.

## Discussion

The findings of our study showed that the majority of patients who underwent aortic valve replacement had significant differences in the ultrasonic B-line count. The number of B-lines increased significantly with the severity of the NYHA functional class, E/E’ and pro-BNP. The invasive LVEDP was elevated in all patients. This was expected to help elucidate whether we were late in deciding on intervention.

The recommendation of aortic valve replacement for aortic stenosis is strong in patients with symptomatic high-gradient AS (class I) and weaker for asymptomatic patients with specific risk factors (class IIb) [4, 6]. Intervention was considered to be effective for asymptomatic patients with high-gradient AS and normal left ventricle function (EF > 55%). Patients tolerated exercise if the procedural risk was low and one of the following parameters was present [4, 6]: very severe aortic stenosis (mean pressure gradient ≥ 60 mmHg or maximum velocity ≥ 5 m/s), severe aortic valve calcification and maximum velocity progression ≥ 0.3 m/s per year according to cardiac computed tomography, and markedly elevated BNP levels (> 3× age- and sex-corrected normal range) verified by multiple tests and without clear explanation.

Detecting ultrasonic B-lines with lung ultrasound is highly sensitive and specific for detecting elevated NT-proBNP [11] and elevated LVEDP and appears superior to classical echocardiographic strategies [12] in pulmonary oedema. In a study of 75 consecutive patients with aortic stenosis, Szabó et al. reported that evaluating B-lines is a simple, highly possible procedure for diagnosing pulmonary congestion in this population [13]. The B-line counts correlate well with the haemodynamic changes caused by AS and patient functional status [13]. Sonographic pulmonary congestion has important prognostic value, as patients with AS who undergo TAVI have a greater chance of negative outcomes if they have a high B-line count [13]. This study revealed severe B-line counts in 27% of the population at presentation and moderate B-line counts in 68% of patients who underwent TAVI. The B line density correlated with the pro-BNP level and the E/E’ ratio, consistent with the findings of previous studies [12]. We did not find a significant correlation with other diastolic dysfunction parameters (E/A, DT, or LAVI) in the aortic stenosis population. Therefore, B-lines in patients with AS might indicate the need for TAVI.

Hubert et al. demonstrated the ability of B-lines to identify elevated.

LVEDP in comparison with echocardiographic protocols. The authors measured the LVEDP and B-lines in patients who underwent coronary angiography. They found that the group with elevated LVEDP (≥ 20 mmHg) had significantly more B-lines in total [17].

Consistent with the findings of previous studies, patients with AS-related pulmonary congestion had a significantly greater number of B-lines. Additionally, patients with 30 or more B-lines have a significantly greater risk of heart failure-related events and death [13]. As we chose in the present study, ≥ 30 B-lines at 28 scanning sites indicated severe congestion [18]. Discharge of patients with acute heart failure and B-lines ≥ 30, irrespective of heart failure aetiology and left ventricle EF status, is an independent predictor of outcome [19]. The use of B-lines via lung ultrasound is a promising method for managing AS because it can help establish symptomatic status in this population. This is particularly important because patients with AS often have a sedentary lifestyle and a high prevalence of comorbidities, restricting physical activity, which can make it challenging to determine their symptomatic status [19]. Although syncope and angina are easily detectable symptoms, heart failure can be subclinical with no symptoms on mild exertion. Therefore, there is a rationale for using lung ultrasound to detect HF early.

A long asymptomatic period occurs during the natural progression of aortic stenosis. Disease progression to symptoms is inevitable, but the compelling question in asymptomatic patients is when to replace the aortic valve. In elderly patients, symptom status may be unclear and difficult to ascertain due to comorbidities or mobility impairment, some of which may be due to sedentary behaviour in asymptomatic patients [20]. In asymptomatic sedentary patients, exercise testing is suggested for select patients with asymptomatic severe AS to confirm asymptomatic status. Exercise testing is particularly helpful when a patient’s level of physical activity is low or only vaguely reported. Patients with severe AS who develop classical symptoms of AS (e.g., exertional dyspnoea) during exercise testing should be regarded as symptomatic even if their clinical history is unclear [6]. The echocardiography-based method CAIMAN-ECHO score is used to improve the prognosis of patients with asymptomatic significant aortic stenosis [21]. The method consists of calculating the calcium score of the aortic valve, the inappropriate left ventricular mass, and the peak gradient across the aortic valve [21]. In addition, Monin et al. included sex, BNP level, and peak aortic jet velocity in a scoring system for asymptomatic severe AS patients, and this system was useful for predicting the midterm risk of death and AVR [22].

Lung ultrasonic B-line assessment might be advantageous for patients with asymptomatic severe or moderate AS. Adding LUS examination to regular TTE might improve the risk categorization of patients with AS. LV injury, mitral valve and LA dysfunction, and diastolic dysfunction result in pulmonary congestion and, consequently, in clinically symptomatic heart failure. Hence, early detection of pulmonary congestion via LUS leads to improved early diagnostic accuracy. In addition, early pulmonary oedema assessment might improve the timing of valve intervention and aid in heart failure management.

### Limitations

This was a single-centre study with an acceptable sample size, but it would be preferable to confirm these findings in larger multicentre studies. The standard gold standard parameter for measuring pulmonary oedema is LVEDP, although some studies consider the mean left atrial pressure as providing a better evaluation of pulmonary congestion [23]. Nonetheless, instead of enrolling patients with atrial fibrillation where left atrial pressure utility is doubtful, LVEDP was chosen as the reference B-line because it can stem from a noncardiac source. Although patients with a high risk of false-positive lung ultrasonography results (such as those with pulmonary fibrosis) were excluded from the study, it is still possible that some of the patients included in this prospective analysis had ultrasonic B-lines that were not related to cardiac issues. Nonetheless, even with these possible measurement limitations, LUS showed outstanding diagnostic performance for high LVEDP.

## Conclusion

Patients with echocardiographic severe AS present late for TAVI, as most of our patients had elevated proBNP and high b-line counts, and all had elevated LVEDP. Evaluating B-lines by lung ultrasound is a simple, highly achievable method for detecting pulmonary oedema in AS patients. The number of B-line artefacts was maintained at E/E’, as was the functional status of the patients. All patients had elevated LVDEPs that correlated with E/E’.

## Data Availability

The datasets used and/or analysed during the current study are available from the corresponding author upon reasonable request.
